# Perceived Strain Due to COVID-19-Related Restrictions Mediates the Effect of Social Needs and Fear of Missing Out on the Risk of a Problematic Use of Social Networks

**DOI:** 10.3389/fpsyt.2021.623099

**Published:** 2021-04-23

**Authors:** Elisa Wegmann, Annika Brandtner, Matthias Brand

**Affiliations:** ^1^General Psychology: Cognition and Center for Behavioral Addiction Research (CeBAR), University of Duisburg-Essen, Duisburg, Germany; ^2^Erwin L. Hahn Institute for Magnetic Resonance Imaging, Essen, Germany

**Keywords:** COVID-19, coronavirus, social media addiction, internet addiction, addictive disorders, fear of missing out, need to belong, coping

## Abstract

The occurrence of the COVID-19-virus led to drastic short-term measures to reduce its spread and influence. Regulations such as “physical distancing,” mentioned as “social distancing,” and the closure of public facilities during the lockdown could be perceived as burdensome especially by individuals who feel a strong need for social exchange and belonging. These components such as need to belong and the fear of missing out also play a major role in the development and maintenance of a problematic use of social networks. Researchers have argued recently that an increase of addictive (online) behaviors may be a likely consequence of subjectively experienced restrictions in the context of the COVID-19 pandemic. The current study investigates the interplay of perceived strain due to COVID-19-related restrictions and the fear of missing out (FoMO) as well as of symptoms of problematic social-networks use. We hypothesized that perceived strain due to COVID-19-related restrictions mediates the effect of specific predisposing variables related to social needs on the symptom severity of a problematic use. To assess the perceived strain due to COVID-19-related restrictions, we developed a specific questionnaire asking for perceived COVID-19-related strain in several domains of everyday-life. An exploratory factor analysis identified five factors: perceived strain related to restrictions of (1) social contacts, (2) travel, (3) childcare, (4) work, and (5) own health. In a sample of 719 German participants and data collection during the first COVID-19 lockdown (March 30th until April 3rd 2020), a structural equation model was calculated showing that higher levels of need to belong and FoMO increase perceived COVID-19-related strain, which is related to symptoms of a problematic social-networks use. The effect of need to belong on problematic social-networks use is mediated by experienced COVID-19-related strain and FoMO-online. Even if the use of social networks is not pathological *per se*, it may be associated with suffering for a vulnerable part of users. We conclude that specific needs and fear-associated predisposing variables contribute to experiencing physical distance and other pandemic-related restrictions as more stressful, which may increase problematic social-networks use and potentially other addictive behaviors as well in the context of the COVID-19-related lockdown.

## Introduction

In 2020, the coronavirus disease, (COVID-19) an infection leading to acute respiratory syndromes, has emerged. In December 2019, the first outbreak of this disease was reported in Wuhan, China, and due to the massive spread across the entire globe, on March 11, 2020, the World Health Organization (WHO) declared the pandemic due to the coronavirus. In order to prohibit the spread and prevent further infections and deaths, governments of many countries imposed unexpectedly drastic changes in societal, cultural, professional, and social life domains. These restrictions include, among others, the temporary closure of public faculties such as schools and kindergartens, the closure of shops, restaurants, and museums, the cancellation of cultural and sporting events, the short-term closure of borders and the issuing of travel warnings as well as the request to cover mouth and nose in public. One of the most important restrictions is the strategy of “social distancing,” often also mentioned as “spatial distancing” or “physical distancing,” which—in addition to create safe, physical distance between people—mainly includes the restrictions of social contacts in real life and to stay at home. This form of self-isolation and contact restrictions seems to be a massive burden, especially for individuals with a strong need for social exchange and belonging. In this context, the WHO as well as several scientists have declared that the usage of digital communication and information technologies could be a good way to stay in touch with family members, friends, and colleagues, and that it may help to maintain a form of social exchange and connectedness with others (1, 2). The use of social networks and other digital online communication applications such as WhatsApp and Facebook therefore play an important role since they allow the exchange and communication with others, the sharing of information, pictures, and videos, and provide further entertainment opportunities during a time when staying at home is the most effective way to break chains of infection (3, 4). Accordingly, Dong et al. (5) and Nimrod (6) illustrate that there was an increase of Internet use in general as well as of social networks, even in the elder generations during the COVID-19 pandemic. Reasons for this increase in Internet use, social networks, and online games could, besides the effect of staying socially connected and feeling entertained, lie in the reductions of stress and unpleasant feelings that could have emerged as a result of physical isolation. Hence, the use of the Internet might be a welcome and functional coping strategy to escape pandemic-associated problems and difficult thoughts for some (1, 7–9).

However, researchers also warn of possible risks regarding social networks and Internet usage not only, but especially during the pandemic (1, 8). While an increase in psychopathological symptoms can generally be observed during the COVID-19 pandemic (5, 10), studies show that the frequent use of the Internet and social networks in particular seems to be associated with mental health problems (11, 12). Rolland et al. (13) as well as Sun et al. (14) demonstrate that addiction-related habits such as eating high caloric food, alcohol consumption, tobacco use, and screen time related to an addictive Internet use have risen. This illustrates the association between mental health issues and the problematic use of the Internet during the COVID-19 pandemic and gives reason to investigate the psychological mechanisms that might make individuals prone to suffer from problematic social-networks use during this time. Researching this question, it is particularly important to take situational circumstances and the perceived strain due to the COVID-19 related restrictions into account.

### Problematic Use of Social Networks and Theoretical Framework Models

As already mentioned, even if the use of social networks and online communication applications offers many advantages and positive aspects, especially for staying in contact with others during the COVID-19 pandemic, there are also individuals reporting negative consequences due to the excessive use of social networks. These reports are part of an ongoing debate regarding the problematic use of social networks which is often defined as “being overly concerned about social networking sites, to be driven by a strong motivation to log on or to use social networking sites and to devote so much time and effort” [(15), p. 4045], whereby individuals experience a diminished control, negative consequences, and impairments in daily life due to the use of these applications (16, 17). The problematic use of social networks has been described as addictive use of social networks or social-networks-use disorder based on the definition of the already classified gaming disorder as disorder due to addictive behaviors in the ICD-11 (18, 19). In addition, researchers discuss whether the problematic use of social networks could be considered as “other specified disorders due to addictive behaviors (coded as 6C5Y) in the ICD-11. Here, Brand et al. (16) argue that three meta-level criteria have to be fulfilled which should be considered as guidelines and which include (1) the scientific evidence for clinical relevance, (2) the theoretical embedding, and (3) the empirical evidence for underlying mechanisms. In the current study, theoretical frameworks of addiction research have been used as basis for deriving the research questions, which will now be described.

The I-PACE model by Brand et al. (20) and its updated version (21) summarizes different theoretical assumptions of addiction research, for example the dual-process approach of addiction (22) and incentive neural sensitization processes (23). Basing on this, the I-PACE model provides a theoretical approach to understand and investigate the process of the development and maintenance of an addictive behavior. One key assumption of this model is the interaction of predisposing factors and affective and cognitive components leading to the continued use of specific online applications or showing a specific behavior. It has been outlined that motives, psychopathological characteristics, personality aspects, and temporal features affect the perception of specific situational features (e.g., mood, stress perception, environmental components). These factors may interact with affective processes (e.g., cue reactivity, craving), internet-related cognitive biases as well as impairments in (specific) inhibitory control and executive functions. Based on conditioned learning processes and reinforcement mechanisms, this may result in the experience of gratification and/or compensation. The constant cycle as part of the addiction process thus forms the basis for the repeated execution of the behavior, but also for the experience of limited control or even a loss of control [for a more detailed description, see (21)]. The overview by Wegmann and Brand (18) picks up key assumptions of the I-PACE model and specifies it for the problematic use of social networks. The authors argue that the use of social networks is mainly associated with psychosocial characteristics that determine either a fear-driven/compensation-seeking approach or a reward-driven approach to use social networks excessively (18). As such, a high need to belong, need for social exchange, perceived social support, and social anxiety depict main motivators that drive behavior in order to experience gratification or compensatory effects due to the usage. As online applications mainly focus on the exchange with other users by creating feelings of social connectedness, psychosocial characteristics and social needs are especially important factors which could *per se* result in a higher risk of an uncontrolled social-networks use. An interaction with specific reinforcement mechanisms such as reductions of fear of missing out and social isolation, or the satisfaction of social needs, could further accelerate the tendency to develop problematic social-networks use. Moreover, Tonioni et al. (24) highlight that a problematic use is also associated with communicative insecurity and a higher need of social support, wherefore it could be argued that on the one hand this is not experienced in real life or on the other hand it is the result of a dysfunctional coping strategy that is related with a higher risk of a problematic use as well [e.g., (25)]. Empirical evidence already illustrates the association of need to belong, social anxiety, and perceived social support related to the problematic use of social networks [e.g., (18, 26–30)].

In addition, research also highlights that the fear that others have more rewarding experiences without oneself, referred to as fear of missing out (FoMO), is an additional key component of a problematic use [e.g., (31–34)]. More precisely, Wegmann et al. (35) differentiate between a general trait-FoMO as a predisposing factor and online-specific state-FoMO as an internet-related cognitive bias where the latter mediates the effect of trait-FoMO on the symptom severity of a problematic social-networks use. To our best knowledge, further studies investigating the mediation effect on social needs such as need to belong and trait-FoMO on the symptom severity have been missing. In addition to the specific predisposing factors and reinforcing mechanisms such as state-FoMO, the I-PACE model by Brand et al. (21) also explores that besides affective and cognitive components, situational aspects play an important role in the understanding of an addictive behavior. It could be argued that the experienced strain due to the social restrictions during the COVID-19 pandemic are such situational aspects which affect the relationship between predisposing factors on the risk of a problematic social-networks use. Therefore, it seems to be important to better understand the interplay of these components in the development and maintenance of a problematic social-networks use.

### Aim of the Current Study

In the current study, we investigated the relevance of subjectively perceived strain due to COVID-19-related restrictions for the development and maintenance of a problematic use of social networks. Several researchers argue that the increase of addiction-related habits such as the increase of the symptom severity of a problematic social-networks use may be a likely consequence of the experienced restrictions in the context of the COVID-19 pandemic. Therefore, we developed a specific questionnaire asking for perceived COVID-19-related strain in several domains of daily life.

**Figure 1:**
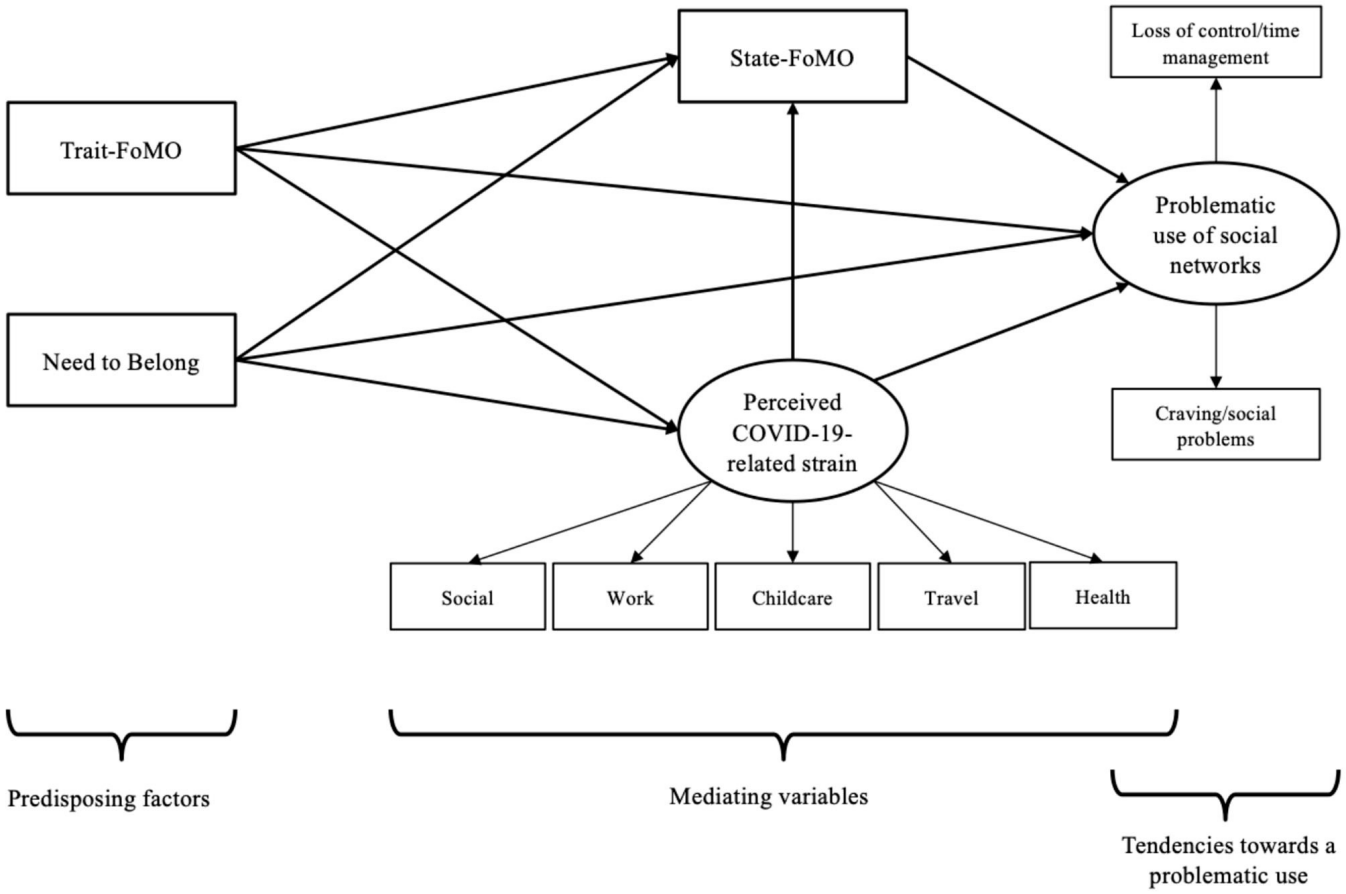
The hypothesized model for analyzing the suggested direct and indirect effects including the latent dimensions of perceived strain due to COVID-19 related restrictions and problematic use of social networks. The figure shows the predisposing factors, the mediator variables, and the dependent variable. The directions of the hypothesized effects are illustrated by the arrows, which also symbolize the direct effects. The rectangles represent the manifest variables, depicted by the subscales of the questionnaires used. The ellipses illustrate the latent dimensions, which are created by the specific manifest variables.

Based on the aforementioned theoretical considerations, we argue that social needs such as need to belong and trait-FoMO are important predisposing factors contributing to the symptom severity of a problematic use of social networks, and that this relationship is mediated by internet-related cognitive biases such as state-FoMO. Considering the situation of the COVID-19-related lockdown, we hypothesized that individuals with high social needs are somewhat deprived in the fulfillment of these needs and therefore experience higher strains due to the COVID-19-related restrictions such as “social distancing” and self-isolation. Experiencing the COVID-19-related restrictions as more burdensome might lead to higher state-FoMO, because the missing opportunity to satisfy social needs by the physical contact to beloved ones might evoke the fear to miss out what they do online. This might cause a more intense use of social networks which could result in a problematic behavior. Therefore, we investigated the interplay of perceived strain due to Covid-19-related restrictions and the fear of missing out in the online world as well as of symptoms of problematic use of social networks. We hypothesized that perceived strain due to Covid-19-related restrictions mediates the effect of specific predisposing variables related to social exchange and social needs on the symptom severity of a problematic use of social networks. The theoretically argued relationships and mediating effects are illustrated in Figure 1.

## Methods

### Participants and Recruitment

Data was collected using a comprehensive online survey which was hosted at University of Duisburg-Essen using the survey software LimeSurvey®. Study participants were recruited and incentivized by an access panel in Germany. Two first screen out questions ensured that participants regularly used a smartphone and that they used communication applications such as Facebook, Twitter, Instagram, or messengers such as WhatsApp. If these queries were answered in the negative, participants were informed that they were not eligible for the study. The survey was online March 30th until April 3rd 2020, right after the “Law to protect the population in the event of an epidemic situation of national importance” came into force in Germany on March 27, 2020. After this survey, participants have been invited to take part in a second survey 4 weeks later, however, this was not part of the current research question. The study was approved by an ethics committee of University Duisburg-Essen.

After careless responder analysis using long-string method, even-odd method, and the investigation of irrational responding times, the final sample consisted of 719 participants (347 females, 48.3%), with a mean age of 50.11 (*SD* = 12.29), ranging from 18 to 79 years. 57.2% of the sample were employees, 19.3% pensioners, 7.5% officials, 6.0% self-employed, and the rest indicated being students, looking for work or other. Participants reported to have used their smartphones averagely 115.72 min (*SD* = 127.03) during the past seven days and to have utilized social networks and messengers for 9.82 years (*SD* = 4.95). Additionally, participants were asked how many minutes they used specific applications per day in February 2020 and during the last 7 days in order to investigate if using times differed. For descriptive results including paired *t*-tests, see Table 1. Gender differences in using times during the last 7 days were identified for Instagram, Smartphone usage, Telephony, and WhatsApp. In all cases, female participants reported significantly higher usage times (*p* ≤ 0.037). In February 2020, only Instagram and WhatsApp were used significantly longer by females (*p* ≤ 0.031).

**Table 1:** Means *(M)* and standard deviations *(SD)* of using times per different social networks and online communication applications as well as the smartphone and Internet use in general.

**Application**	***N***	**February 2020**		**Last 7 days**		***t*-test**
		***M***	***SD***	***M***	***SD***	
Internet	719	189.71	165.32	200.70	157.75	0.003
Smartphone	719	105.09	125.95	115.72	127.03	0.002
Telephony	719	27.62	64.47	35.00	70.88	<0.001
WhatsApp	684	38.26	75.22	42.70	71.47	0.010
Facebook	290	45.48	85.32	50.39	78.32	0.172
Facebook Messenger	215	22.79	108.42	15.03	27.64	0.228
Instagram	162	39.41	106.71	35.25	44.87	0.523
Skype	80	22.81	60.18	29.62	53.28	0.218
Twitter	62	43.85	91.62	43.15	83.05	0.844
Threema	37	24.11	56.80	21.78	47.21	0.718
iMessage	36	22.69	82.68	15.33	34.04	0.385
Snapchat	24	51.75	181.29	46.21	140.10	0.522

*All using times are indicated in minutes per day. M = mean, SD = standard deviation*.

### Instruments

#### Need to Belong

To assess a general need to belong, the 10-item Need to Belong Scale (36) was used. As there is no validated German version so far, the questionnaire was translated into German and re-translated into English by four independent research assistants. Exemplary items are “I don't like being alone.” or “It bothers me a lot when I am not involved in the planning of others.” which are answered on a five-point Likert scale (1 = *completely disagree* to 5 = *completely agree*). The internal consistency of the questionnaire in this sample reached Cronbach's α = 0.798.

#### Fear of Missing Out

To measure FoMO as trait- and state-variable, we utilized the scale introduced by Wegmann et al. (35). This version was modified and extended with online-specific items basing on the original 10-item Fear of Missing Out Scale (37). Wegmann et al. (35) detected a two-factor structure of their 12-item version with one factor depicting *trait-FoMO* (five items; e.g., “I feel insecure when I do not know what my friends are up to.”) and the other factor representing *state (online) FoMO* (seven items; e.g., “I am continually online, to not miss out on anything.”). Items are answered on a five-point Likert scale (1 = *completely disagree* to 5 = *completely agree*). The internal consistency of the questionnaire in this sample reached Cronbach's α = 0.812 for trait-FoMO and Cronbach's α = 0.848 for state-FoMO.

#### Problematic Use of Social Networks

Tendencies toward symptoms of problematic use of social networks were assessed using a modified version of the short Internet Addiction Test (s-IAT-com) for online-communication applications (38) which bases on the s-IAT as introduced by Pawlikowski et al. (39). The two factors of the 12-item questionnaire are represented by six items each. Items of the factor *loss of control/time management* (e.g., “How often do you find that you have used online communication applications for longer than you intended?”) and the factor *craving/social problems* (e.g., “How often do you react evasively or defensively when someone asks you what you do online?”) were answered on a five-point Likert scale (1 = *never* to 5 = *very often*). The internal consistency of the questionnaire in this sample reached Cronbach's α = 0.911.

#### Perceived COVID-19-Related Strain

To operationalize the perceived strain during the Covid-19-associated restrictions, a total of 16 items were developed on the basis of consideration. These items asked for how much several restrictions that were initiated to prohibit the spread of the pandemic were perceived as burdensome. Among the restrictions and consequences due to the lockdown were the recommendation to work from home, the cancellation of orders, or the closure of public places and borders. Each of the restrictions and consequences were rated on a 5-point Likert scale (1 = *not at all burdensome* to 5 = *very burdensome*).

To explore the factorial structure of these 16 items, an exploratory factor analysis (EFA) with principal axis factoring, promax rotation, and parallel analysis by Horn (40) was conducted with the data of the current sample. During this procedure, items were discarded on the basis of poor combinations of primary and secondary factor loadings. This procedure resulted in a stable twelve-item and five-factor solution. The factors that were extracted could thematically be classified as experienced strain due to social contact restrictions (three items), restrictions in the working context (three items), childcare restrictions (two items), travel restrictions (two items) and health issues (two items), see Table 2.

**Table 2:** Item factor loadings, means, standard deviations, and Cronbach's α of the subscales of the questionnaire assessing perceived strain due to COVID-19-related restrictions.

**Items**	***M (SD)***	**Factor**				
		**1**	**2**	**3**	**4**	**5**
**Social**						
Avoidance of social contacts	3.21 (1.23)	**0.983**	−0.023	−0.028	−0.098	−0.013
Restrictions in public life	3.07 (1.14)	**0.797**	0.024	−0.009	0.062	0.028
Prohibition of contact to others than family	3.26 (1.37)	**0.675**	0.008	0.059	0.081	−0.017
**Reliability α = 0.860**						
**Work**						
Initiation of short-term working	1.81 (1.29)	−0.014	**0.783**	0.036	−0.046	−0.069
Cancellation of orders	1.95 (1.24)	0.007	**0.760**	−0.058	0.051	−0.087
Existential livelihood/unemployment	1.87 (1.18)	0.015	**0.659**	0.024	−0.010	0.190
**Reliability α = 0.774**						
**Childcare**						
Closure of playgrounds	1.72 (1.19)	−0.019	−0.017	**0.899**	0.002	0.027
Closure of schools and childcare facilities	2.04 (1.42)	0.031	0.016	**0.791**	−0.006	−0.044
**Reliability α = 0.825**						
**Travel**						
Closure of borders	2.24 (1.41)	0.030	−0.021	0.008	**0.815**	0.082
Travel warnings/cancellations of holiday trips	2.80 (1.48)	−0.013	0.016	−0.011	**0.796**	−0.085
**Reliability α = 0.784**						
**Health**						
Own illness	1.79 (1.11)	−0.027	0.035	0.011	0.015	**0.815**
Own previous illness	1.98 (1.22)	0.022	−0.052	−0.025	−0.023	**0.738**
**Reliability α = 0.745**						

*M = mean, SD = standard deviation*.

*The primary factor loadings are highlighted in bold*.

### Statistical Analysis

The statistical analyses were carried out with SPSS 26.0 for Mac. There were no missing data. We calculated Pearson correlations testing the bivariate correlations between two manifest variables. The structural equation model analyses were computed with Mplus 8 (41). For evaluating the model fit of the model, standard criteria were used: standardized root mean square residual (SRMR; values < 0.08 indicate a good fit with the data), comparative fit indices (CFI/TLI; values > 0.90 indicate an acceptable and values > 0.95 indicate a good fit with the data), and root mean square error of approximation (RMSEA; values between 0.08 and 0.10 indicate an acceptable and values < 0.08 indicate a good fit with the data) (42, 43). All variables for the structural equation model were required to correlate with each other (44).

## Results

### Descriptive Values and Correlation Analysis

The descriptive values of the s-IAT and the scores of the questionnaires as well as the bivariate correlations are shown in Table 3. The results illustrated significant correlations between all variables applied. We found no significant relationship between factor *Health* of the COVID-19 related strain and the symptom severity of problematic social-networks use. Therefore, we excluded the factor in the structural equation model. In addition, based on the reported cut-off scores by Pawlikowski et al. (39), 52 participants (7.23% of the sample) indicated a problematic use of social networks (cut-off score ≥ 31), and 22 participants (3.01% of the sample) a pathological use (cut-off score ≥ 38).

**Table 3:** Descriptive statistics and bivariate correlations between the symptom severity of a problematic use of social networks and the applied scales.

	***M (SD)***	**2**	**3**	**4**	**5**	**6**	**7**	**8**	**9**	**10**	**11**	**12**
1. s-IAT-com sum score	20.18 (7.14)	0.948^**^	0.934^**^	0.276^**^	0.427^**^	0.510^**^	0.219^**^	0.261^**^	0.253^**^	0.192^**^	0.079^*^	0.339^**^
2. s-IAT-com loss of control	10.88 (4.02)		0.772^**^	0.287^**^	0.387^**^	0.480^**^	0.238^**^	0.256^**^	0.261^**^	0.198^**^	0.051	0.342^**^
3. s-IAT-com craving/social problems	9.30 (3.56)			0.229^**^	0.420^**^	0.479^**^	0.171^**^	0.235^**^	0.213^**^	0.162^**^	0.101^**^	0.294^**^
4. Need to Belong	3.17 (0.65)				0.382^**^	0.343^**^	0.458^**^	0.143^**^	0.233^**^	0.191^**^	0.065	0.382^**^
5. Trait-FoMO	1.98 (0.79)					0.587^**^	0.228^**^	0.162^**^	0.168^**^	0.094^**^	0.025	0.236^**^
6. State-FoMO	1.86 (0.75)						0.219^**^	0.148^**^	0.149^**^	0.145^**^	0.008	0.234^**^
7. COVID-19-related strain *Social*	3.18 (1.11)							0.234^**^	0.349^**^	0.405^**^	0.042	0.729^**^
8. COVID-19-related strain *Work*	1.88 (1.01)								0.276^**^	0.237^**^	0.140^**^	0.653^**^
9. COVID-19–related strain *Childcare*	1.88 (1.21)									0.268^**^	0.005	0.620^**^
10. COVID-19-related strain *Travel*	2.52 (1.31)										0.002	0.646^**^
11. COVID-19-related strain *Health*	1.89 (1.04)											0.322^**^
12. COVID-19-related strain *Overall*	2.31 (0.69)											

*M = mean, SD = standard deviation*,

* *p ≤ 0.050*,

** *p ≤ 0.010*.

### Structural Equation Modeling

**Figure 2:**
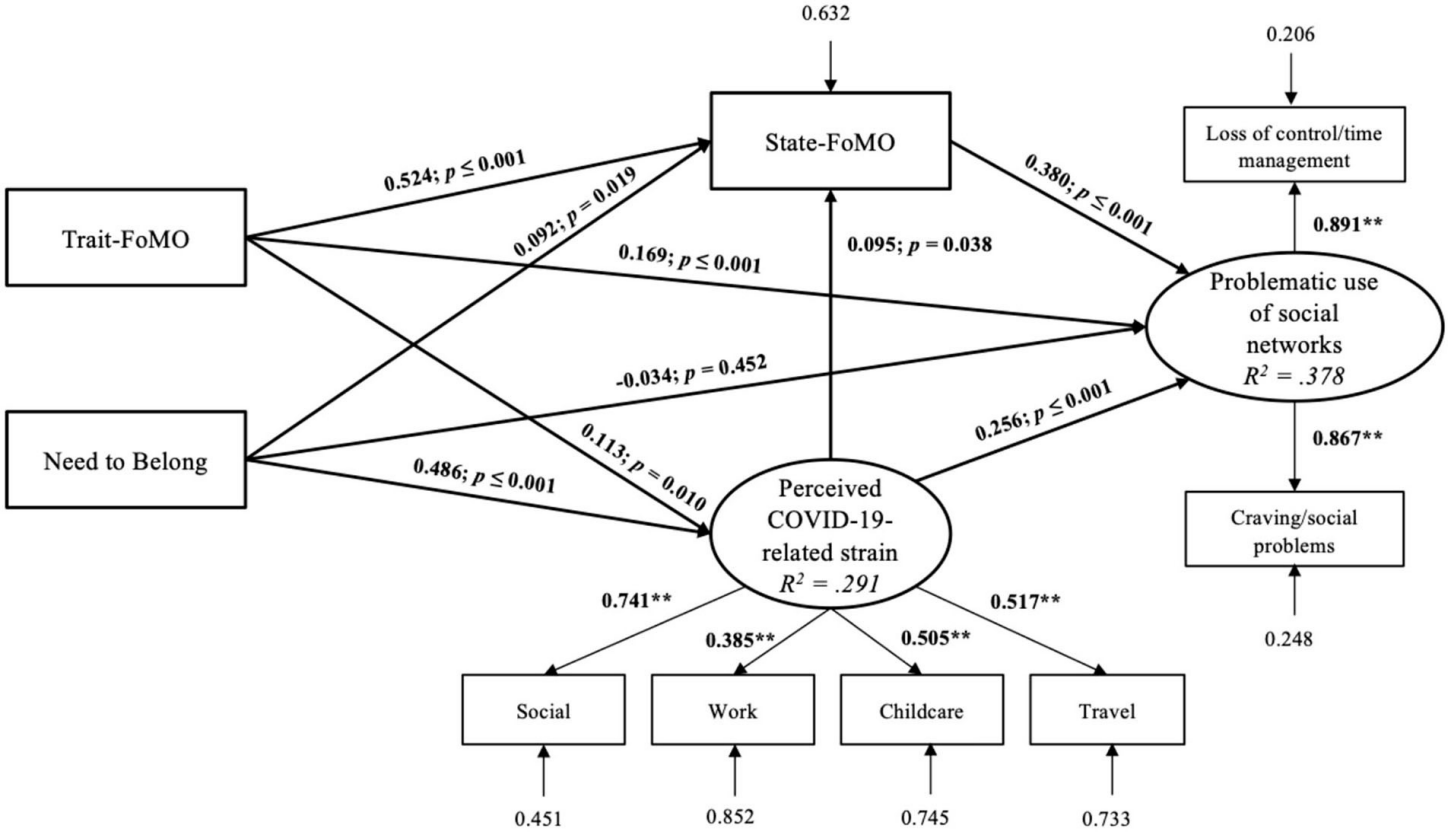
Results of the structural equation model with problematic use of social networks as dependent variable including factor loadings on the described latent dimensions and the accompanying β-weights, *p*-values, and residuals. The directions of the hypothesized effects are illustrated by the arrows. The rectangles represent the manifest variables, depicted by the subscales of the questionnaires used. The ellipses illustrate the latent dimensions, which are created by the specific manifest variables.

The proposed model on latent dimension with symptom severity of problematic social-networks use showed a good fit with the data (RMSEA = 0.069, *p* = 0.018; CFI = 0.962; TLI = 0.933; SRMR = 0.040). Overall, 37.8% of the variance of the symptom severity could be explained by the proposed direct and indirect effects. The latent dimensions *problematic use of social networks* and *perceived COVID-19-related strain* were well-represented by the manifest variables. The results illustrate that the perceived COVID-19-related strain as well as trait-FoMO and state-FoMO showed a direct effect on the symptom severity. The COVID-19-related strain, trait-FoMO, and need to belong also had a direct effect on state-FoMO, and in addition, trait-FoMO and need to belong showed a direct effect on COVID-19-related strain as well. We found significant indirect effects; the effect of trait-FoMO on symptom severity was mediated by state-FoMO (β = 0.199, SE = 0.025, *p* ≤ 0.001) and by COVID-19-related strain (β = 0.029, SE = 0.013, *p* = 0.027), but not the path of both (β = 0.004, SE = 0.003, *p* = 0.106). The effect of COVID-19-related strain on symptom severity was also mediated by state-FoMO (β = 0.026, SE = 0.017, *p* = 0.036). Even if we could not illustrate a direct effect of need to belong on the symptom severity, we found that the effect was mediated by state-FoMO (β = 0.035*, SE* = 0.016, *p* = 0.026), COVID-19-related strain (β = 0.124, SE = 0.029, *p* ≤ 0.001), and by both, COVID-19-related strain, and state-FoMO indicating a full-mediation effect (β = 0.018, SE = 0.009, *p* = 0.040). The structural equation model with factor loadings and β-weights are represented in Figure 2. For an overview, all coefficients for direct and indirect effects of the SEM are also summarized in Table 4.

**Table 4:** Overview of the standardized coefficients illustrating the direct and indirect effects in the SEM.

	**Effects**	**β**	**SE**	***p***
Direct effects	Trait-FoMO—State-FoMO	0.524	0.029	<0.001
	Trait-FoMO—Strain	0.113	0.044	0.010
	Trait-FoMO—Problematic Use	0.169	0.043	<0.001
	Need to Belong—State-FoMO	0.092	0.039	0.019
	Need to Belong—Strain	0.486	0.041	<0.001
	Need to Belong—Problematic Use	−0.034	0.045	0.452
	Strain—State-FoMO	0.095	0.046	0.038
	Strain—Problematic Use	0.256	0.054	<0.001
	State-FoMO—Problematic Use	0.380	0.041	<0.001
Indirect effects	Trait-FoMO—State-FoMO—Problematic Use	0.199	0.025	<0.001
	Trait-FoMO—Strain—Problematic Use	0.029	0.013	0.027
	Trait-FoMO—Strain—State-FoMO—Problematic Use	0.004	0.003	0.106
	Need to Belong—State-FoMO—Problematic Use	0.035	0.016	0.026
	Need to Belong—Strain—Problematic Use	0.124	0.029	<0.001
	Need to Belong—Strain—State-FoMO—Problematic Use	0.018	0.009	0.040
	Strain—State-FoMO—Problematic Use	0.036	0.017	0.036

## Discussion

### General Discussion of the Results

In the current study, the effect of subjectively perceived strain due to COVID-19-related restrictions on the symptom severity of a problematic use of social networks has been investigated. We also examined if the perceived strain as well as state-FoMO mediate the effect of social characteristics needs such as trait-FoMO and need to belong on the development and maintenance of the problematic behavior. We therefore developed a specific questionnaire assessing perceived COVID-19-related strain in several domains of everyday life. The results of an exploratory factor analysis identified a five-factor solution illustrating strain related to social contact restrictions, restrictions in the working context, childcare restrictions, travel restrictions, and health issues. Pearson correlation analyses showed that the strain due to COVID-19-related restrictions was associated with the tendency of a problematic use of social networks as well as with need to belong, trait-FoMO, and state-FoMO with small to medium effect sizes. As the only factor, the strain related to health issues showed no consistent correlations with the symptom severity and some of the other constructs. Rather than measuring perceived strain due to own health issues, it could be assumed that this factor is more related to a general fear or anxiety due to or of the COVID-19 virus itself [cf., (45)].

The structural equation model also outlines that the perceived strain could be identified as potential accelerating factor of the problematic social-networks use. The analysis demonstrates that trait-FoMO had a direct effect on the symptom severity, but that there had been a partial mediation effect by the COVID-19-related strain as well. There was no direct effect of need to belong on problematic use of social networks which indicates that higher social needs do not lead automatically to habitually using social networks and developing problematic behaviors. Rather, the results showed a full mediation effect of need to belong on symptom severity due to, among other, the perceived strain. These results highlight that individual characteristics and social needs such as the necessity for social connectedness and an alongside fear to miss out what friends and acquaintances experience, do not *per se* and isolated predict the problematic use of social networks. Instead, these findings assign a prominent role to the perceived strain or stress due to situational circumstances when investigating determining factors for an enhanced risk for a problematic use. In the I-PACE model, Brand et al. (20) also stress out that the subjective perception of situational factors, which are related to perceived stress and abnormal mood, could result in a higher risk of using the Internet dysfunctionally, or as a strategy to cope with stress. This is in line with the model of Compensatory Internet Use by Kardefelt-Winther (46) reflecting that using the Internet or social networks as for compensation and particularly as coping strategy could result in a problematic behavior. The author emphasizes the importance of considering environmental factors as complementary components that might trigger coping mechanisms or a problematic behavior. Referring to this theory, the COVID-19-related restrictions could be such environmental factors, especially since research already outlines that the Internet in general and social networks in specific represent coping strategies during the COVID-19 pandemic (1, 9, 14). Accordingly, individual differences in responding to situational circumstances and restrictions could trigger subjectively perceived strain or stress, affecting the relationship between personality aspects, and social needs, which does not determine, but may enhance the risk to use social networks problematically.

Besides the situational factors, the findings also show that internet-related cognitive biases as proposed in the I-PACE model (20, 21) are additional reinforcing factors leading to a higher risk of a problematic use of social networks. The effect of need to belong and trait-FoMO on the symptom severity was mediated by state-FoMO. It highlights that it is worth not investigating persons' core characteristics solely, but also specific cognitions and further reinforcing processes. Nevertheless, the results are in line with previous studies showing that social needs, psychosocial characteristics and emotional impairments (e.g., need for exchange, need to belong, perceived social support) are related to a problematic social-networks use in general [e.g., (18, 24, 26, 28, 29, 47)]. It has also been demonstrated that FoMO is a risk factor and in addition mediates the effect of psychopathological symptoms on symptom severity [e.g., (48–50)]. Comparable with Wegmann et al. (35), we would like to make this association even more precisely by differentiating between the general fear that others have rewarding experiences while being absent and the online specific state. Therefore, the current findings expand the empirical evidence since they highlight the outstanding position of online-specific FoMO as reinforcing mechanism of the relationship between predisposing factors and a problematic behavior. This process is part of the fear-driven/compensation-seeking hypothesis by Wegmann and Brand (18). The hypothesis outlines that high social needs and the expectancies to reduce feelings of social isolation and FoMO by using social networks may drive problematic behavior. The additional mediation effects of the structural equation model in this study indeed show that the effect of social needs on the symptom severity was mediated by the perceived COVID-19-related strain which was mediated by state-FoMO as well. The considerations of the fear-driven hypothesis can thus be expanded by that additional external strain may result in higher fear of missing out online, which could enhance the risk of a problematic use. This path is also postulated by the I-PACE model (20, 21) showing that a person's core characteristics (i.e., a tendency for social needs) impact on the situational perception of external triggers (i.e., COVID-19-related strain), which affect specific cognitions such as internet-related cognitive biases (i.e., state-FoMO), and then enhance the chance to experience a diminished control over the behavior.

The result that social needs may not automatically be associated with the problematic use of social networks impact the derivation of prevention and practical implications. It means that specific cognitions, but in particular fears, coping strategies, expectancies, the experience of gratification and compensation, as well as emotion regulation should be focused. Individuals who do not expect to experience gratification and compensation, to feel better or experience pleasure, and to deal with stress, negative emotions and fear exclusively online have a lower risk of developing a problematic use. Therefore, it is crucial to learn, posses, and be able to apply functional coping strategies and emotion regulation skills. The environmental and situational factors may facilitate these processes or—as it is the case of the restriction during the COVID-19 pandemic—complicate them. The gratification of social needs such as feelings of belonging or physical as well as real-life social contact are extremely limited by the strategy of social distancing. In a situation that may already be perceived as very stressful, further restrictions such as the closure of sport or leisure facilities makes it even more difficult to apply further coping strategies. The use of social networks or playing online games is an approach to deal with individual needs and fears, but since they carry the risk to be used dysfunctionally, the establishment of further strategies is of great importance. With regard to our results, we think that it could be of particular interest to address the perception of strain and stress as preventive mechanisms. The functional handling of perceived strain and stress may reduce the risk of an addictive behavior. Concurrently, it also includes the consideration which alternative coping strategies can be used to satisfy needs for connectedness and belonging while maintaining the strategy of social distancing, and to use social networks in this context functionally without using it as the only strategy for social well-being. Central aspects in cultivating resilience to distress admit the COVID-19 pandemic refer to the creation of meaning, for example by taking goal- and value-oriented activities (51) such as pursuing hobbies, physical activity, and a daily routine (1, 52, 53). Going outdoors, but also just looking outside has a potential to reduce, for example, depressive symptoms (52). Other possible indoor-activities that have an individual stress-reducing effect and help to handle one's emotions might include reading, writing, meditation and mindfulness exercises, and openly communicating arising emotions to family members or close friends (1). Further, some authors argue that strengthening a feeling of human interconnectedness and positive reappraisal/reframing of the current situation might soothe a feeling of social desertion (51, 53). Respective strategies that have been proposed include acceptance-based coping and loving-kindness practices (51). More specific propositions that directly target the use of social networks address the reductions of screen time per day, including the regulation of one's own as well of children's usage (1).

Finally, there are some limitations to be mentioned. In the current study, we developed a new questionnaire assessing perceived strain due to COVID-19-related restrictions. This self-report needs further validations, especially because it was constructed during a time period which was very dynamic and contained a high uncertainty in Germany. We highly recommend to apply this questionnaire in further studies during the COVID-19 pandemic and additionally to investigate convergent and divergent validity such as general fear and stress perception related to COVID-19 [see also (45, 54, 55)]. We also consider it important to discuss the sample of the current study in relation to previous findings. The average age represents a middle age about 50 years, which differs significantly from previous research mainly investigating student samples with an average age of 30 years. Since empirical studies already outlined that the problematic use of social networks could mainly be found in younger age or even in middle age (56–58), the lower symptom severity is not unexpected. However, it has to be considered that an increase of social-networks use and the Internet in general could be observed in elderly generations during the COVID-19 pandemic as well (6). Lastly, in the current study, we used cross-sectional data which is another important aspect to bear in mind. Even if this snapshot makes an important contribution to gaining knowledge of the potential development and maintenance of a problematic use of social networks during the COVID-19 pandemic, longitudinal studies are particularly important in this context. This would allow assessing the effect of long-term consequences of the perceived restrictions and the pandemic circumstances on individual well-being in general as well as on the social-networks use specifically.

## Conclusion

Investigating the psychological effects on individual well-being during the COVID-19 pandemic is an important task in psychological research. This includes the question of how certain behaviors such as the use of social networks, but also potential addictive tendencies may change. The present study contributes to this question by examining the subjectively perceived strain due to the COVID-19-related restrictions in several life domains in relation to the problematic use of social networks and social needs. The results showed that for the development and maintenance of a problematic use, the effect of social needs should not be investigated in isolation, since internet-related cognitive biases and situational factors such as perceived strain may represent additional accelerating mechanisms. We conclude that social needs and fear-associated predisposing variables contribute to experiencing physical distance and other pandemic-related restrictions as more stressful, which may then increase problematic social-networks use in the context of the COVID-19-related lockdown. Reducing the subjectively experienced strains related to the COVID-19-related restrictions by clarification of facts and the importance of such restrictions and by considering stress-reduction techniques and mindfulness may be helpful for both dealing with the restrictions and preventing problematic use of social networks.

## Data Availability Statement

The raw data supporting the conclusions of this article will be made available by the authors, without undue reservation.

## Ethics Statement

The studies involving human participants were reviewed and approved by the ethics committee of the division of Computer Science and Applied Cognitive Sciences at the Faculty of Engineering, University of Duisburg-Essen. The ethics committee waived the requirement of written informed consent for participation.

## Author Contributions

EW, AB, and MB contributed to conception and design of the study as well as performed and interpreted the statistical analysis. EW and AB organized the database. EW wrote the first draft of the manuscript. AB and MB wrote sections, edited, and revised the manuscript critically. All authors read and approved the submitted version.

## Conflict of Interest

The authors declare that the research was conducted in the absence of any commercial or financial relationships that could be construed as a potential conflict of interest.
